# Preparation and NMR spectra of four isomeric diformyl[2.2]paracyclophanes (cyclophanes 66)

**DOI:** 10.3762/bjoc.6.104

**Published:** 2010-09-29

**Authors:** Ina Dix, Henning Hopf, Thota B N Satyanarayana, Ludger Ernst

**Affiliations:** 1 Institut für Organische Chemie, Technische Universität Braunschweig, Hagenring 30, D-38106 Braunschweig, Germany; 2 Department of Organic Chemistry, Indian Institute of Science, Bangalore, 560012, Karnataka, India; 3 NMR-Laboratorium der Chemischen Institute, Technische Universität Braunschweig, Hagenring 30, D-38106 Braunschweig, Germany

**Keywords:** cyclophanes, diformyl[2.2]paracyclophanes, layered compounds, NMR spectroscopy, structure assignment

## Abstract

Four isomeric dialdehydes **4**, readily available from cycloaddition of propiolic aldehyde (**2**) to 1,2,4,5-hexatetraene (**1**), were separated by chromatography and recrystallization, and were characterized by their spectroscopic data. The individual isomers can now be easily identified from their ^1^H NMR spectra even if only one of them is present.

## Introduction

**Scheme 1:**
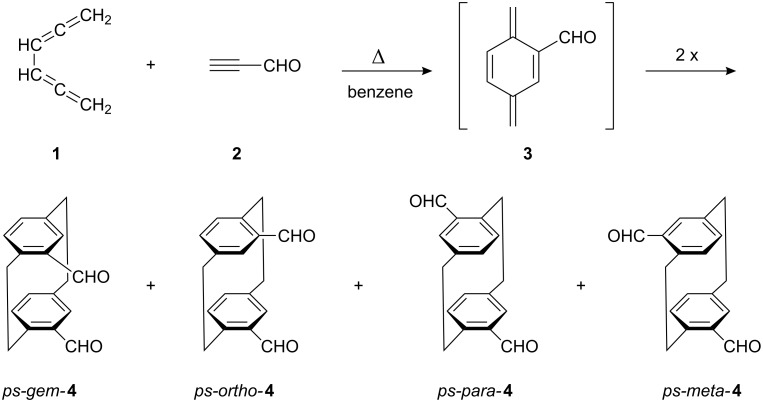
Preparation of the four [2.2]paracyclophane dialdehydes **4** by cycloaddition (ps - pseudo).

Previously, we reported that the [2 + 4] cycloaddition of 1,2,4,5-hexatetraene (**1**) to propiolic aldehyde (**2**) produced a mixture of four [2.2]paracyclophane dialdehydes **4**. This result is in agreement with the generation of the *p*-xylylene intermediate **3** in the first step of the reaction, which can dimerize by four different modes (Scheme 1) [2]. Depending on the amounts and the quality of the solutions containing the tetraene **1** and the dienophile **2**, more than 60 g of the dialdehyde mixture **4** can be obtained in a single run, corresponding to maximum yields of 46%. Both starting compounds are very reactive and can decompose, even under refrigeration.

Although we have separated the four isomers and used them many times for the preparation of numerous [2.2]paracyclophane derivatives (inter alia annelated derivatives [2], metal complexes [3–5], diethynyl derivatives [6–7] and preparation of ligands for chiral reagents [8–9]), we have neither described the separation of these versatile starting materials nor explained their spectroscopic and analytical data in full. With this short communication, we would finally like to remedy this omission, in particular since these compounds are also beginning to attract the attention of other research groups [6–7,10–13].

## Results and Discussion

### Separation of the diformyl[2.2]paracyclophanes 4

We have separated the isomer mixture **4** by different methods. The easiest way is by middle pressure liquid chromatography (MPLC), which readily affords all isomers in gram amounts. To obtain larger amounts of specific isomers, e.g. the pseudo-*para* compound, it might be advisable to use the combination of column chromatography and MPLC or recrystallization described in the experimental section. In alcoholic solvents, *pseudo*-*para*-**4** is the least soluble of all isomers and may hence be easily separated.

#### NMR spectra of the diformyl[2.2]paracyclophanes 4

As the ^1^H and ^13^C NMR spectra of [2.2]paracyclophane-4-carbaldehyde have previously been fully assigned and, hence, the influence of the substituent upon the ^1^H and ^13^C NMR chemical shifts of all positions of [2.2]paracyclophane are known [14], the assignment of the dicarbaldehyde isomers could, in principle, be derived from the comparison of their experimental chemical shifts with those calculated by assuming substituent chemical shift (SCS) additivity. However, there is no certainty that such an assumption is justified, as it is difficult to judge whether steric and/or electronic substituent interactions could cause significant deviations from SCS additivity. Furthermore, the SCSs exerted by the formyl group upon the nuclei of the distant ring are relatively small, |Δδ_H_| < 0.11 ppm, |Δδ_C_| < 0.88 ppm. We therefore decided to seek independent experimental proof for the four isomeric structures.

**Scheme 2:**
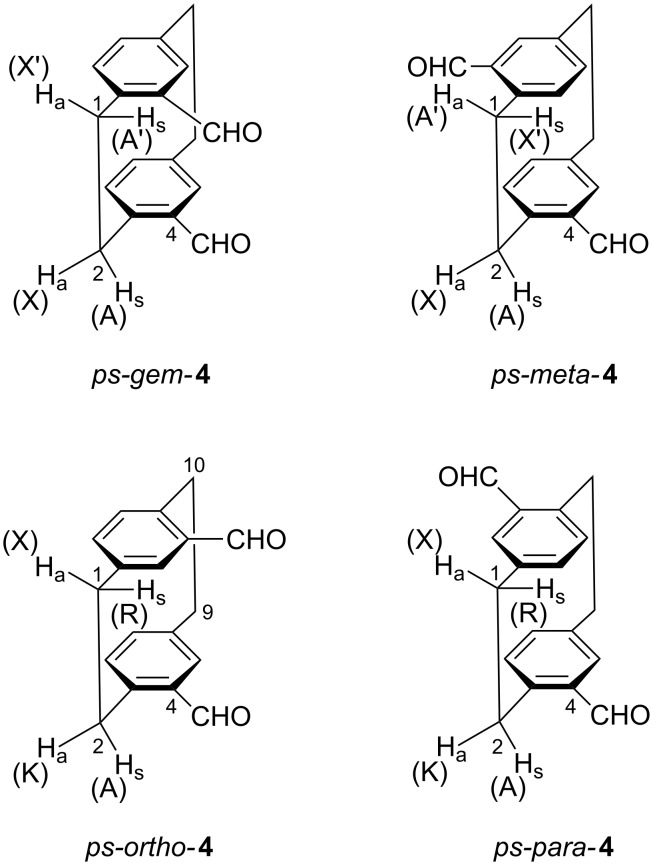
Spin systems of the CH_2_CH_2_ protons in the isomeric dialdehydes **4**. Protons at C-9 and C-10 in *ps-gem*- and *ps-meta*-**4** form YY´ZZ´ spin systems, which need not be considered here. Protons at C-9 and C-10 in *ps-ortho*- and *ps-para*-**4** form AKRX spin systems equivalent to those of the C-1 and C-2 protons.

For this purpose, consideration of their symmetry is helpful. The *ps-gem* and the *ps-meta* isomer both have a symmetry element (plane and two-fold axis, respectively), which causes each CH_2_CH_2_ bridge to have only two different ^1^H chemical shifts. The protons of the bridge close to the formyl substituents form an AA´XX´ spin system, see Scheme 2. These protons are labelled H_a_ or H_s_ depending on whether they are *anti* or *syn* with respect to the 4-CHO group.

**Figure 1:**
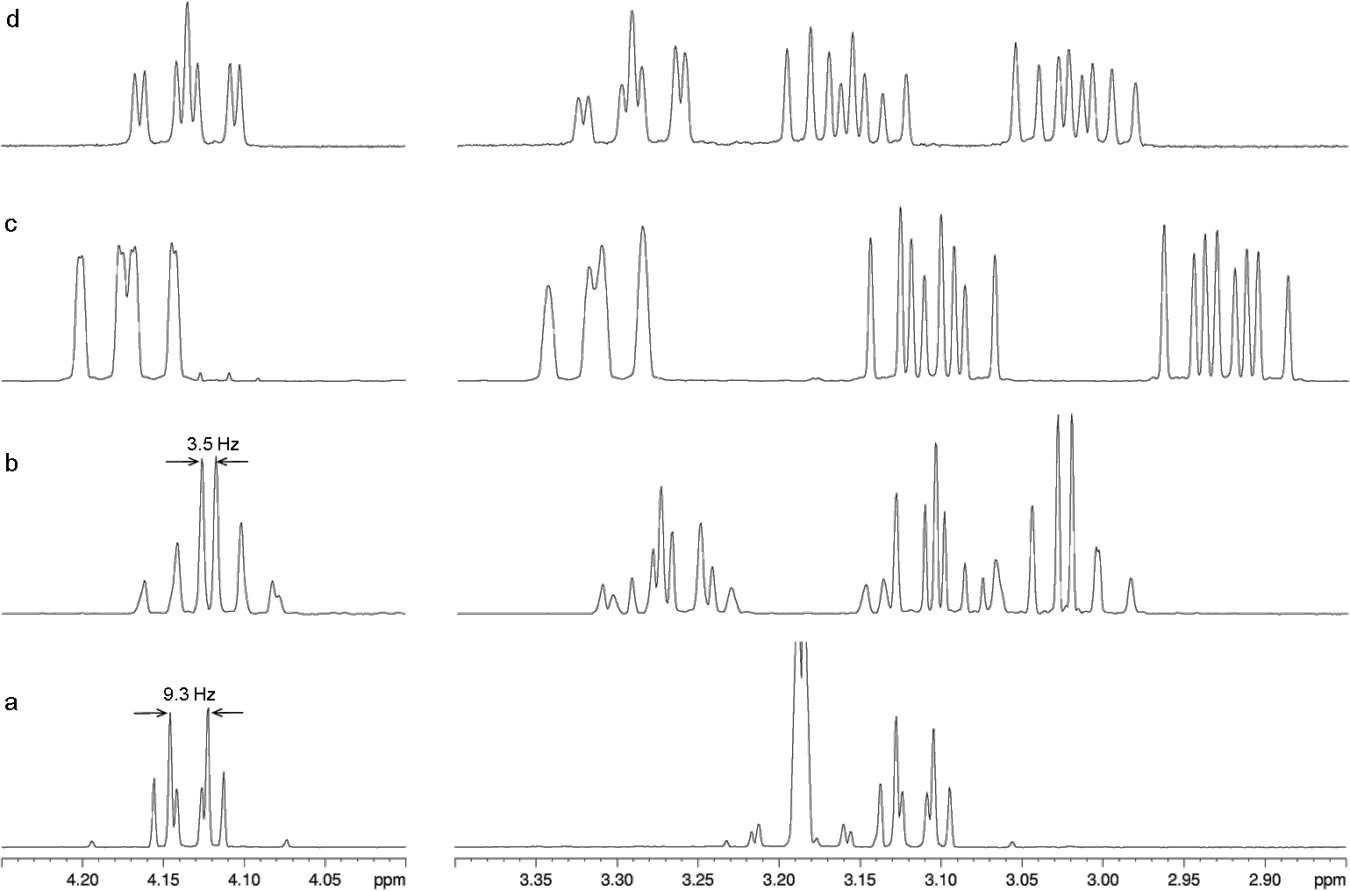
CH_2_CH_2_ regions of the 600 MHz ^1^H NMR spectra of the isomeric dialdehydes **4**, a: *ps-gem*, b: *ps-meta*, c: *ps-ortho*, d: *ps-para* isomer.

The most characteristic and most easily recognized feature of an AA´XX´ spectrum is the distance *N*, defined as |*J*(AX)+*J*(AX´)|. In the *ps-gem* isomer, *N* equals |*J*_gem_+*J*_trans_| with *J*_gem_ ≈ –13 Hz and *J*_trans_ ≈ +4 Hz [14]_._ Hence, *N* is expected to be near 9 Hz. In the *ps-meta* isomer, *N* equals |*J*_gem_+*J*_cis_| with *J*_gem_ as above and *J*_cis_ ≈ +10 Hz, hence *N* ≈ 3 Hz. Spectra a and b in Figure 1 have *N* = 9.3 and 3.5 Hz, respectively, and therefore correspond to the *ps-gem* and the *ps-meta* isomer in this order. The *ps-ortho* and the *ps-para* isomer have a 2-fold axis and a centre of symmetry, respectively, which render the two bridges in each compound equivalent. So each of these isomers gives rise to a single AKRX spin system for their bridges, see spectra c and d in Figure 1.

**Scheme 3:**
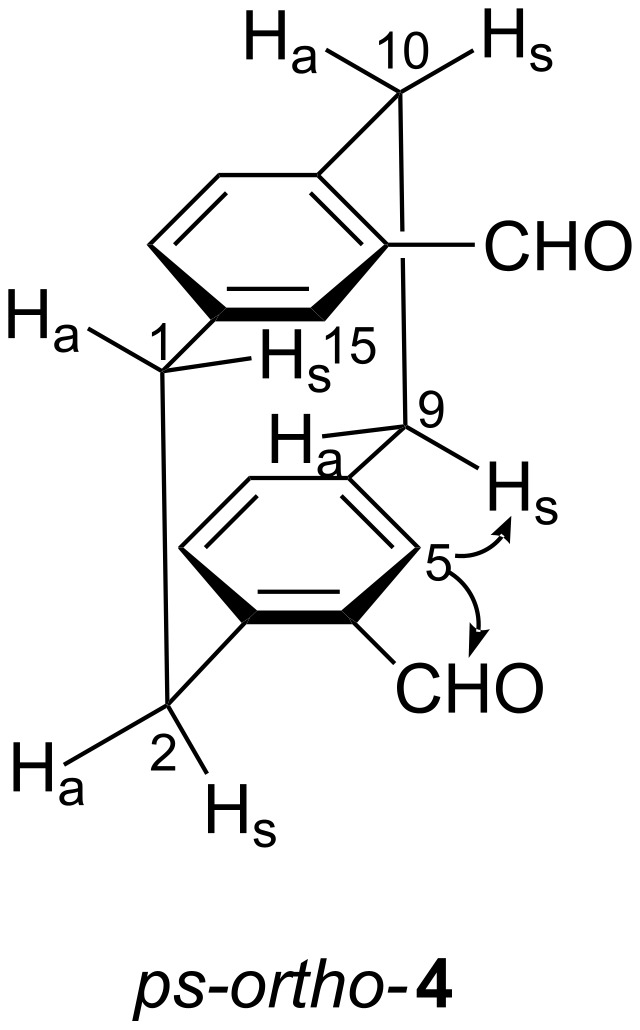
NOEs observed for *ps-*ortho-**4** when the 5-H resonance is irradiated.

The distinction of the *ps-ortho* from the *ps-para* isomer is made possible with the help of an NOE experiment. In the isomer belonging to the spectrum shown in Figure 1c, the multiplet at δ = 3.11 ppm experiences a strong NOE when the resonance of 5-H (*ortho* to the 4-CHO group) is saturated, and vice versa. Its coupling constants show that the 3.11 ppm multiplet belongs to 9-H_s_ (*cis* to the deshielded proton 10-H_s_, which itself is *syn* to the second formyl group, see Scheme 3).

This proves the *ps-ortho* arrangement of the two aldehyde groups. Then, by default, the spectrum in Figure 1d is that of *ps-para*-**4**. The ^1^H and ^13^C NMR data of the isomers are given in Table 1 and Table 2.

**Table 1:** ^1^H NMR data of the isomeric dialdehydes (CDCl_3_/TMS).

	*ps-gem*-**4**^a,b^	*ps-ortho*-**4**^a,c^	*ps-meta*-**4**^a,d^	*ps-para*-**4**^a,e^

5-H (d)	6.99	6.87	7.04	7.05
7-H (dd)	6.75	6.79 (ddd)	6.65	6.64
8-H (d)	6.66	6.61	6.49	6.53
CHO (s)	9.81	9.82	9.98	9.94
1-H_a_	3.12 (m)	3.32 (ddd)	3.03 (m)	3.29 (ddd)
1-H_s_	4.13 (m)	3.11 (ddd)	4.12 (m)	3.16 (ddd)
2-H_a_		2.93 (ddd)		3.02 (ddd)
2-H_s_		4.17 (ddd)		4.14 (ddd)
9-H_a_	≈3.19 (m)		3.27 (m)	
9-H_s_	≈3.19 (m)		3.10 (m)	

^a^ *J*(5,7) = 2.0, *J*(7,8) = 7.8 Hz. ^b^ *N* = |*J*(1a,1s) + *J*(1a,2s)| = 9.3 Hz. ^c^ *J*(1a,1s) = (–)13.3 Hz, *J*(1a,2a) = 10.1 Hz, *J*(1a,2s) = 1.3 Hz, *J*(1s,2a) = 7.4 Hz, *J*(1s,2s) = 10.0 Hz, *J*(2a,2s) = (–)13.1 Hz, *J*(7,9a) = 0.8 Hz. ^d^ *N* = |*J*(1a,1s) + *J*(1a,2a)| = 3.5 Hz. ^e^ *J*(1a,1s) = (–)13.2 Hz, *J*(1a,2a) = 10.7 Hz, *J*(1a,2s) = 2.4 Hz, *J*(1s,2a) = 5.8 Hz, *J*(1s,2s) = 10.4 Hz, *J*(2a,2s) = (–)13.1 Hz.

**Table 2:** ^13^C NMR data of the isomeric dialdehydes (CDCl_3_, δ = 77.01 ppm).

	*ps-gem*-**4**	*ps-ortho*-**4**	*ps-meta*-**4**	*ps-para*-**4**

CHO	191.36	192.00	191.68	191.92
C-3 (C_q_)	142.75	142.52	143.19	142.92
C-4 (C_q_)	137.00	136.41	136.94	136.79
C-5 (CH)	134.63	136.16	136.12	136.55
C-6 (C_q_)	140.42	140.69	140.57	140.54
C-7 (CH)	137.94	137.86	137.47	136.98
C-8 (CH)	136.07	136.21	134.89	135.24
C-1 (CH_2_)	31.91	34.19	33.71	34.36
C-2 (CH_2_)		33.87		32.81
C-9 (CH_2_)	34.72		34.66	

The question of SCS additivity posed above can now be answered as follows. In the ^1^H NMR spectra, additivity is satisfied with, in general, |Σ SCS_obs_–Σ SCS_calc_| ≤ 0.06 ppm, except for 2-H_a_ and 2-H_s_ in *ps-meta*-**4** where one finds 0.11 and 0.17 ppm, respectively. In the ^13^C NMR spectra, SCS additivity is less strict, namely |Σ SCS_obs_–Σ SCS_calc_| ≤ 0.7 ppm, except for C-2, C-4 and C-5 in *ps-gem*-**4** with 1.2, 1.1 and 0.9 ppm, respectively. We also find that SCS additivity was distinctly better in the previously studied analogous bis(dimethoxycarbonyl)[2.2]paracyclophanes [15] compared to the present dialdehydes. This may be attributed to the larger magnitudes of the SCS of the aldehyde compared to those of the methyl ester functional group.

## Conclusion

Although the present method of preparing the dialdehydes **4** involves extensive separation work (chromatography and recrystallization), it offers certain advantages compared to alternative (more specific) routes [10–13]. It is much shorter than many of the alternatives, which involve the rather expensive starting material, [2.2]paracyclophane. This method also provides four different substitution patterns in one step, two of which are interesting chiral derivatives (pseudo-*ortho*- and pseudo-*meta*-**4**). This leads directly to derivatives with a preparatively very useful functional group. The four individual isomers can now be easily identified, even if only one isomer is present, because each one shows a characteristic multiplet for the proton 2-H_s_ at δ_H_ = 4.1–4.2 ppm: i.e., *ps-gem*-**4** and *ps-meta*-**4** give an AA´XX´ pattern with *N* = 9.3 and 3.5 Hz, respectively; to a good approximation, *ps-ortho*-**4** has a dd pattern with *J* = 13 and 10 Hz; and *ps-para*-**4** displays a ddd pattern with the two inner lines coinciding (rel. intensities 1:1:1:2:1:1:1, *J* = 13, 10.5 and 2.5 Hz). Another characteristic is the small chemical shift difference δ_5-H_−δ_7-H_ of 0.08 ppm for *ps-ortho*-**4**, whereas this difference is 0.24–0.41 ppm for all other isomers.

## Experimental

### Separation of the isomers 4

After the cycloaddition has been carried out [3], the precipitate formed was removed by filtration. The mother liquor was concentrated to half its volume and the solution stored in the refrigerator overnight. The newly formed crop was filtered off and united with the first precipitate. The combined solid reaction products were treated with chloroform in a Soxhlet extractor to remove any polymeric material produced. The solvent was removed and the residual solid could be directly separated by MPLC chromatography (silica gel) with dichloromethane/ethyl acetate (98:2, v/v) to provide the analytically pure isomers **4** (product ratio: *pseudo*-*gem*- : *pseudo*-*ortho*- : *pseudo*-*meta*- : *pseudo*-*para*-**4**: 1.8 : 1.1 : 1.0 : 2.8); (*R*_f_ = 0.47, 0.55, 0.60, 0.63). To reduce the separation effort, the above product mixture can be enriched first by standard silica gel chromatography with dichloromethane/ethyl acetate (98:2, v/v). This procedure provided three fractions: a fast moving one consisting of *pseudo*-*para*- and *pseudo*-*meta*-**4**, a middle fraction containing all four isomers, and a third fraction consisting of the *pseudo*-*ortho*- and the *pseudo*-*geminal* isomers. If necessary, this process can be repeated, thus allowing separation of the four dialdehydes into two fractions each containing two isomers.

These may then be subjected to MPLC chromatography or repeated normal pressure column chromatography, allowing, for example, the separation of pure *pseudo*-*ortho*-**4**. Alternatively, the *pseudo*-*meta*/*para*-mixture could be purified by recrystallization from methanol, in which the *pseudo*-*para* isomer is only poorly soluble. On refluxing this mixture in methanol, followed by separation of the precipitate, practically pure pseudo-*para* compound was obtained. To remove the last traces of isomeric impurities from *pseudo*-*para*-**4**, it was recrystallized from *n*-butanol.

### Spectroscopic properties

The NMR spectra were measured at 600 MHz (^1^H, CDCl_3_, TMS, δ = 0.00 ppm) and at 151 MHz (^13^C, CDCl_3_, δ = 77.01 ppm) on a Bruker Avance II 600 instrument. Assignments were carried out using H,H-COSY, H,C-HSQC, H,C-HMBC and NOEDIF techniques.

#### 4,15-Diformyl[2.2]paracyclophane (*ps*-*gem*-4)

^1^H NMR: see Table 1. ^13^C NMR: see Table 2. IR (KBr): 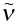 = 3029 (w), 2926 (w), 1676 (vs), 1588 (m), 1482 (m), 1225 (m), 1141 (m), 720 cm^–1^ (m). UV (acetonitrile): λ_max_ (lg ε) = 214 (4.57), 262 (4.13), 320 nm (3.41). MS (70 eV): *m*/*z* (%) = 264 (100) [M^+^], 236 (45), 207 (11), 132 (87), 104 (95).

#### 4,16-Diformyl[2.2]paracyclophane (*ps*-*ortho*-4)

^1^H NMR: see Table 1. ^13^C NMR: see Table 2. IR (KBr): 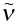 = 3028 (vw), 2927 (w), 1680 (vs), 1648 (m), 1552 (m), 1232 cm^–1^ (m). UV (acetonitrile): λ_max_ (lg ε) = 216 (4.53), 296 nm (3.95). – MS (70 eV): *m*/*z* (%) = 264 (100) [M^+^], 132 (98), 104 (92).

#### 4,12-Diformyl[2.2]paracyclophane (*ps*-*para*-4)

^1^H NMR: see Table 1. ^13^C NMR: see Table 2. IR (KBr): 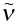 = 3013 (w), 2933 (w), 1684 and 1673 (vs), 1586 (m), 1483 (m), 720 cm^–1^ (m). UV (acetonitrile): λ_max_ (lg ε) = 214 (4.55), 264 (4.16), 320 nm (3.42). MS (70 eV): *m*/*z* (%) = 264 (100) [M^+^], 132 (94), 104 (100).

#### 4,13-Diformyl[2.2]paracyclophane (*ps*-*meta*-4)

^1^H NMR: see Table 1. ^13^C NMR: see Table 2. IR (KBr): 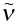 = 2936 (w), 1678 (vs), 1588 (m), 1487 (w), 1229 (m), 1141 (w), 900 (w), 778 (m), 719 cm^–1^ (m). UV (acetonitrile): λ_max_ (lg ε) = 214 (4.54), 296 nm (3.95). MS (70 eV): *m*/*z* (%) = 264 (82) [M^+^], 236 (17), 207 (9), 132 (66), 104 (100).

## References

[1] Wullbrandt D, Hopf H, Jones P G (2010). Eur J Org Chem.

[2] Aly A A, Hopf H, Ernst L (2000). Eur J Org Chem.

[3] Hopf H, Raulfs F-W, Schomburg D (1986). Tetrahedron.

[4] Hopf H, Raulfs F-W (1985). Isr J Chem.

[5] El-Tamany S, Raulfs F-W, Hopf H (1983). Angew Chem, Int Ed Engl.

[6] Hopf H, Hinrichs H, Dix I, Bondarenko L (2004). Synthesis.

[7] Hopf H, Dix I (2006). Synlett.

[8] Hillmer J (1991).

[9] Laue T (1991).

[10] Clément S, Guyard L, Knorr M, Däschlein C, Strohmann C (2009). Acta Crystallogr, Sect E: Struct Rep Online.

[11] Morisaki Y, Lin L, Chujo Y (2009). J Polym Sci, Part A: Polym Chem.

[12] Nandivada H, Chen H-Y, Bondarenko L, Lahann J (2006). Angew Chem, Int Ed.

[13] Hopf H (2008). Angew Chem, Int Ed.

[14] Ernst L (1995). Liebigs Ann.

[15] Ernst L (1997). Fresenius' J Anal Chem.

